# Phytochemical Screening, Free Radical Scavenging and *α*-Amylase Inhibitory Activities of Selected Medicinal Plants from Western Nepal

**DOI:** 10.3390/medicines6020070

**Published:** 2019-06-25

**Authors:** Kusum Sai, Rashmi Thapa, Hari Prasad Devkota, Khem Raj Joshi

**Affiliations:** 1 School of Health and Allied Sciences, Faculty of Health Sciences, Pokhara University, Pokhara 33700, Nepal; kusumsai100@gmail.com (K.S.); rasmithp@gmail.com (R.T.); khemraj_pu@yahoo.com (K.R.J.); 2 Graduate School of Pharmaceutical Sciences, Kumamoto University, 5-1 Oehonmachi, Chuo-ku, Kumamoto 862-0973, Japan

**Keywords:** medicinal plants, Nepal, total phenolic content, total flavonoid content, DPPH, *α*-amylase

## Abstract

**Background:** More than 700 plants are reported to be used for medicinal purposes in Nepal; however, many of them are not studied for their scientific evidences. The aims of the present study were the estimation of the total phenolic and flavonoid contents, and the evaluation of the free radical scavenging and α-amylase inhibitory activities of five selected medicinal plants from western Nepal: *Aeschynanthus parviflorus* Wall. (Gesneriaceae), *Buddleja asiatica* Lour. (Loganiaceae), *Carica papaya* L. (Caricaceae), *Drepanostachyum falcatum* (Nees) Keng f. (Gramineae) and *Spondias pinnata* (L. f.) Kurz (Anacardiaceae). **Methods:** The total phenolic content (TPC) and total flavonoid content (TFC) were measured using Folin-Ciocalteu’s phenol reagent and aluminium chloride methods, respectively. A 1,1–diphenyl–2–picrylhydrazyl (DPPH) free radical scavenging assay was used to evaluate the free radical scavenging activity and an α-amylase inhibitory assay was carried out to determine the in vitro antidiabetic activity. **Results:** The phytochemical screening of five hydroalcoholic plant extracts revealed the presence of various secondary metabolites, including alkaloids, flavonoids, reducing sugars, saponins, terpenoids and tannins. The amounts of total phenolics and flavonoids were found to be the highest in *B. asiatica* leaf extract, which also showed the most potent free radical scavenging activity. Extract of *C. papaya* fruits showed the highest α-amylase inhibitory activity, whereas the extracts of *B. asiatica* leaves and *S. pinnata* leaves exhibited moderate activity. **Conclusions:** Some of the medicinal plants selected in this study showed high TPC and TFC values and potent bioactivities. These results may provide the scientific evidences of the traditional uses of these plants. However, further detailed studies on bioactive compounds isolation and identification and evaluation of in vivo pharmacological activities should be performed in future.

## 1. Introduction

In most developing countries, medicinal plants and their products are utilized as important therapeutic agents for treating various diseases [1,2]. Countries in South Asia, including Nepal and India, have a long history, since the Vedic period, of using crude drugs obtained from plant sources as medicine [3]. Nepal is a small landlocked country blessed with a natural and cultural diversity. The biodiversity of Nepal is unique due to its climatic conditions and many isolated topographical locations. More than 700 plants are reported to be used for medicinal purposes in Nepal [1,4,5]. However, these resources have been underutilized, and there is still a huge potential for researchers to explore the floristic and faunal diversity. There are many medicinal herbs yet to be studied for their bioactive chemical constituents and potential therapeutic activities. Although these medicinal plants are used by ethnic people for various purposes, there is very limited information available regarding their mechanisms of action, dose, frequency, side effects and many other factors that otherwise would be very necessary to provide scientific evidences. Thus, in this study, we selected the following five plants, i.e., *Aeschynanthus parviflorus* Wall. (Gesneriaceae), *Buddleja asiatica* Lour. (Loganiaceae), *Carica papaya* L. (Caricaceae), *Drepanostachyum falcatum* (Nees) Keng f. (Gramineae) and *Spondias pinnata* (L. f.) Kurz (Anacardiaceae), from Western Nepal and screened for their phytochemical constituents and biological activities.

*A. parviflorus* is an epiphytic sub-shrub distributed in India, Nepal, Sikkim, Bhutan, Southern China, Burma, Thailand and Vietnam [6]. Root decoction is used for the treatment of fever, and the infusion of leaves is used to treat tonsillitis in Mizoram, India [7]. Plant juice, about 4 teaspoons twice a day, helps to conceive. Powdered leaf, mixed with rice flour, is baked and taken for backaches [4].

*B. asiatica*, also called butterfly bush, is an evergreen shrub endemic to East Asian countries including India and Nepal [8]. In Nepal, the leaves and flowers are used as a religious offering to gods and goddesses. Squeezed leaves are used as a fish poison. The plant juice or paste is used for diarrhea, skin diseases and beverage fermentation [9].

*C. papaya* is a tropical tree, native to central America and now widely cultivated in all tropical and sub-tropical regions for its edible fruits and its latex [10]. The medicinal properties of *C. papaya* are well documented in traditional systems of medicine [11] Latex of papaya is anthelmintic and used for wound healing and skin diseases. Leaf decoction is used for asthma and rheumatism. The unripe fruits are used as a laxative and diuretic [12,13].

*D. falcatum*, also called hill bamboo, is a herb commonly found in the subtropical forest in Nepal, Bhutan and India [14]. This plant has a religious importance in Nepal as it is frequently used in Hindu marriages and house warming rituals. It is also used as an effective soil stabilizer in farming [15]. Young shoots are cooked as vegetable, and the stems are used in making baskets, mats and as fodder [4].

*S. pinnata* is a deciduous tree, 10–15 m tall, indigenous to Southeast Asian countries [16,17]. Different parts of *S. pinnata* (leaves, bark, fruits, and roots) have been used for the treatment of various disorders. For example, the fresh leaves of *S. pinnata* are used to treat gastrointestinal disorders, and the ripe fruits are consumed raw or as juice as a liver tonic and appetizer in Nagaland, India [18]. The bark is used for the treatment of dysentery, muscular rheumatism and diabetes mellitus [19]. The fruits are eaten fresh or pickled. Bark decoction is given for dysentery, gonorrhea and rheumatism [20]. The roots are used for regulating menstruation [21].

The main aims of the present study were the estimation of the total phenolic and flavonoid contents, and the evaluation of the free radical scavenging and *α*-amylase inhibitory activities of these five medicinal plants.

## 2. Materials and Methods

### 2.1. Chemicals

1,1–Diphenyl–2–picrylhydrazyl (DPPH), gallic acid and quercetin were purchased from Wako Pure Chemicals, Osaka, Japan. α-Amylase was obtained from Hi-Media Laboratories, Mumbai, India. Ascorbic acid, aluminium chloride and starch were procured from Qualigens Fine Chemicals, Mumbai, India. Folin-Ciocalteu’s phenol reagent was obtained from Sigma Aldrich, St. Louis, MO, USA.

### 2.2. Plant Materials

Five plant species (Table 1) were collected from different localities of Kaski district, Western Nepal, during the month of August, 2017. The plant species were identified by Dr. Radheshyam Kayastha, Former Professor, Tribhuvan University, Nepal. The voucher specimens were deposited at the Laboratory of Pharmacognosy, Pokhara University, Nepal. Their voucher specimen numbers are given in Table 1.

### 2.3. Extraction

The dried plant samples (30–50 g) were macerated twice with 80% ethanol (1:8 *w*/*v*) for 24 h in a closed vessel with occasional shaking. The filtered extracts were then dried through the use of a rotary evaporator. The percentage yield of the extracts (Table 1) was calculated using the following equation: yield (%) = (weight of extract / weight of dried plant material) × 100 [22].

### 2.4. Phytochemical Screening

The extracts obtained from the selected plant parts were subjected to a preliminary screening to identify the secondary metabolites, using different phytochemical tests [23].

### 2.5. Determination of Total Phenolic Content (TPC)

The TPC of the plant extracts was determined using Folin-Ciocalteu’s phenol reagent method as described by Hazra et al. [24], with a slight modification. In brief, 1 mL of extract (1 mg/mL) was mixed with 5 mL of distilled water and 1 mL of Folin-Ciocalteu’s phenol reagent. After 5 min, 1 mL of 10% (*w*/*v*) sodium carbonate was added and mixed properly. The mixture was allowed to stand for 1 h at room temperature, and then the absorbance was measured at 725 nm using a UV spectrophotometer. The TPC of each extract was calculated using an equation obtained from the standard calibration curve of gallic acid and was expressed in terms of the gallic acid equivalent (mg of GAE/g of extract). All experiments were performed in triplicate.

### 2.6. Determination of Total Flavonoid Content (TFC)

The content of the flavonoids in the plant extracts was performed using the aluminium chloride colorimetric method by Chang et al. [25], with a slight modification. In brief, 1 mL of extract (1 mg/mL) was mixed with 4 mL of distilled water, and 0.3 mL of 5% sodium nitrite was added. After 5 min, 0.3 mL 20% aluminium chloride was added and allowed to stand for 6 min. Then, 2 mL of 1 M sodium hydroxide was added. The mixture was shaken, and the absorbance was measured at 510 nm using a UV spectrophotometer. The TFC of each extract was calculated using an equation obtained from the standard calibration curve of quercetin and was expressed in terms of the quercetin equivalent (mg of quercetin/g of extract). All experiments were performed in triplicate.

### 2.7. Free Radical Scavenging Activity

The antioxidant activity of the plant extracts was determined using the DPPH free radical scavenging assay [26], with a slight modification. Briefly, 2 mL of different concentrations of extract/standard solution were mixed with 2 mL of 60 μM DPPH solution. After mixing, the mixture was incubated for 30 min in dark at room temperature, and the absorbance was measured at 517 nm using a UV spectrophotometer. Ascorbic acid was used as a positive control. The scavenging activity of each sample against the DPPH free radical was calculated using the following equation: Scavenging Activity (%) = [(Ac − As)/Ac] × 100. Where, Ac = Absorbance of the control and As = Absorbance of the sample. A graph was obtained by plotting the scavenging activity (%) against the concentration, and the inhibitory concentration (IC_50_) value was calculated, which is defined as the concentration of the sample required to scavenge 50% of the DPPH free radicals. All experiments were performed in triplicate.

### 2.8. In Vitro α-Amylase Inhibitory Activity

The α-amylase inhibitory assay was performed through the modified starch iodine protocol [27], with a slight modification. In brief, 1 mL of plant extract/standard of different concentrations (0.25 mg/mL, 0.5 mg/mL, 1 mg/mL, and 2 mg/mL) was added to 1 mL of 0.02 M phosphate buffer (pH 6.9 with 0.006 M NaCl) containing α-amylase (1 mg/mL) solution and was be incubated at 37 °C for 10 min. Then, 1 mL of 1% starch solution was added to each test tube. The reaction mixture was then incubated at 37 °C for 1 h. After incubation, the reaction was stopped by adding 0.04 mL of 1 M HCl, followed by the addition of 0.1 mL of 1% iodine reagent. The absorbance was measured at 565 nm using a UV spectrophotometer. The percentage inhibition was calculated by using the following expression: Percentage inhibition = [(As − Ac)/As] × 100. Where, Ac is the absorbance of the control and As is the absorbance of the sample. From these data, a curve was plotted, and the inhibitory concentration (IC_50_) value was calculated, which is defined as the concentration of the samples required for a 50% inhibition of enzyme. All experiments were performed in triplicate.

### 2.9. Statistical Analysis

The results were expressed as the mean ± SD (n = 3). All of the data analyses were carried out using Microsoft Excel 2007.

## 3. Results

### 3.1. Extraction and Phytochemical Screening

The extraction yields of the extracts are given in Table 1. The phytochemical screening of the plant extracts showed the presence of various phytochemicals, such as alkaloids, flavonoids, reducing sugars, saponins, terpenoids and tannins, as shown in Table 2.

### 3.2. Total Phenolic and Flavonoid Contents (TPC and TFC)

The TPC was expressed as the GAE/g of extract using a standard calibration curve of gallic acid (y = 0.0097x + 0.2507, r^2^ = 0.9924). Similarly, The TFC was expressed as the QE/g of extract using a calibration curve of quercetin (y = 0.0004x + 0.1243, r^2^ = 0.995). The amounts TPC and TFC were found to be highest in extracts of *B. asiatica* leaves (127.48 ± 1.58 mg GAE/g, and 648.42 ± 2.88 mg QE/g, respectively), followed by *S. pinnata* leaves (71.50 ± 1.39 mg GAE/g, and 425.08 ± 1.44 mg QE/g, respectively). The *D. falcatum* shoots extract showed the lowest phenolic and flavonoid contents (Table 3).

### 3.3. DPPH Free Radical Scavenging Activity

The results of the DPPH free radical scavenging activities are expressed as IC_50_ (μg/mL) in Table 4. The *B. asiatica* leaf extract exhibited the strongest free radical scavenging activity with an IC_50_ value of 3.04 ± 0.04 μg/mL, followed by the *S. pinnata* leaf extract (IC_50_ = 4.84 ± 0.12 μg/mL), when compared to the positive control, ascorbic acid (IC_50_ = 3.16 ± 0.03 μg/mL).

### 3.4. α-Amylase Inhibitory Activity

The results of the α-amylase inhibitory activity of the extracts are given in Table 4. Among the tested samples, the *C. papaya* fruit extract showed the most potent activity with an IC_50_ value of 0.45 ± 0.02 mg/mL. Similarly, the *B. asiatica* leaves (IC_50_ = 1.59 ± 0.01 mg/mL) and *S. pinnata* leaves (IC_50_ = 2.11 ± 0.01 mg/mL) exhibited a moderate inhibitory activity against the α-amylase enzyme.

## 4. Discussion

Plants contain diverse bioactive compounds, also known as secondary metabolites, which are reported to exhibit various health promoting activities in the human body, including antioxidant activity [28,29]. One such chemical class is phenolic compounds, which are widely distributed in plants and are included as an important part of the human diet owing to their antioxidant and various other medicinal properties [30]. Flavonoids are the largest group of naturally occurring phenolic compounds; they are reported to have various biological activities including antioxidant, antimicrobial, antiulcer, antidiabetic, hepatoprotective and anticarcinogenic activities [31]. In this study, the results showed that *S. pinnata* leaves and *B. asiatica* leaf extracts have a potent antioxidant activity with IC_50_ values of 4.84 ± 0.12 μg/mL and 3.04 ± 0.04 μg/mL respectively, as compared to the positive control, ascorbic acid (IC_50_ = 3.16 ± 0.03 μg/mL). The total phenolic and flavonoid contents were also found to be higher in these extracts (Table 3). Therefore, the strong antioxidant activity of *S. pinnata* and *B. asiatica* leaf extracts can be connected with their high amount of phenolics and flavonoids. This result further supports that the antioxidant activity of these plants is in agreement with their total phenolic and flavonoid contents, as stated in previous studies [24,32]. Phenolic compounds and flavonoids with unsubstituted hydroxyl groups have been recognized as potent free radical scavengers [33,34]

α-Amylase hydrolyses α-linked polysaccharides such as starch and glycogen. [35]. α-Amylase inhibitors, e.g., acarbose and *α*-glucosidase inhibitors e.g., voglibose reduce the postprandial glucose levels by competitively inhibiting these hydrolase enzymes, thus delaying the absorption of glucose [36]. In our study, the α-amylase inhibitory activity of *C. papaya* was found to be higher, among the studied plant samples, with an IC_50_ value of 0.45 ± 0.02 mg/mL. *B. asiatica* leaves (IC_50_ = 1.59 ± 0.01 mg/mL) and *S. pinnata* leaves (IC_50_ = 2.11 ± 0.01 mg/mL) exhibited a moderate activity against α-amylase. However, these results should be compared with positive control e.g., acarbose in future studies. The phytochemical screening of *C. papaya* fruit extract revealed the presence of alkaloid, reducing sugar, saponin and tannin in this study. From previous studies, it is well known that phenolic compounds, including flavonoids and tannins, are useful for the prevention and management of diabetes mellitus [37,38,39]. Recent studies have reported the enzyme inhibitory actions of plant phenolics, with a strong inhibitory effect on α-glucosidase but a mild effect on α-amylase, thus suggesting its use for the treatment and management of diabetes [40]. The inhibition and slowing down of the activity of carbohydrate metabolizing enzymes, such as *α*-amylase or *α*-glucosidase, are referred as potential therapeutic targets in diabetes [41].

There have been some previous studies on the chemical constituents and bioactivities of these selected plants. *B. asiatica* possess different classes of phytochemicals, including flavonoids, sterols, iridoid glucosides, triterpenoids, phenylpropanoid esters and non-phenolic compounds [42]. 6–*O*–(3″, 4″–Dimethoxycinnamoyl) catalpol was isolated from the flowering parts of *B. asiatica* [43]. Similarly, 3,4 dihydroxy phenylethyl alcohol 8–*O*[(4′–*O*-feruoyl)–α–L–rhamnopyranosyl–(1″→3′)–*β*–D-glucopyranosyl–(1‴→6′)]–*β*–D–glucopyranoside was isolated from the leaves [44]. *B. asiatica* has been reported to exhibit different pharmacological activities, including antibacterial, antifungal, antispasmodic, cytotoxic, anti-inflammatory and antihepatotoxic activities [45]. *C. papaya* is well known for its nutritive and medicinal values. The unripe pulp of *C. papaya* contains phytochemicals, such as saponins and cardinolides, as well as minerals, including potassium, sodium, calcium, iron, phosphorus, zinc, copper and magnesium [46]. Similarly, the HPLC profile of *C. papaya* leaves showed flavonols–manghaslin, clitorin, rutin, nicotiflorin, and piperidine alkaloids–carpamic acid, methyl carpamate and carpaine [47]. The aqueous extract of *C. papaya* leaves is reported to exhibit an anti-tumor activity against human lymphocytes and showed immunomodulatory effects [48]. Extensive studies on the chemical isolation and biological activities of *D. falcatum* and *A. parviflorus* have not been reported yet. In this study, a preliminary phytochemical screening of *A. parviflorus* and *D. falcatum* hydroethanolic extracts only showed the presence of terpenoids among the different phytochemicals that were tested. Both plant extracts exhibited a poor free radical scavenging activity and α-amylase inhibitory activity, and they also possess a low quantity of total phenols and flavonoids. *S. pinnata* is known to exhibit various pharmacological activities, such as antimicrobial, antidiabetic, ulcer-protective, anticancer, antidiarrhoeal, anthelmintic, cytotoxic and hepatoprotective activities [49]. The methanol extract of the *S. pinnata* bark showed a promising hypoglycemic activity in normal and alloxan induced diabetic rats [50]. Methyl gallate isolated from the *S. pinnata* bark exhibited an anticancer activity by the induction of a sustained extracellular signal-regulated kinase 1/2 activation and apoptosis in human glioblastoma [51]. A study on the nutraceutical and therapeutic potential of raw *S. pinnata* has also revealed that its fruits are rich in amino acids, calcium, phosphorus, ascorbic acid and malic acid [52]. Further detailed studies on the bioassay-guided isolation and identification of compounds are necessary to identify promising leads for therapeutic applications.

## 5. Conclusions

In this study, five medicinal plants from western Nepal were screened for their phytochemical constituents, and their free radical scavenging and *α*-amylase inhibitory activities. The results concluded that *S. pinnata* and *B. asiatica* leaf extracts were found to possess a strong antioxidant activity and higher contents of total phenol and flavonoid. The *α*-Amylase inhibitory activity of *C. papaya* fruits was found to be higher among the tested plant samples, whereas *B. asiatica* and *S. pinnata* leaves exhibited a moderate activity. Further studies should focus on the bioassay-guided chemical analysis and in-vivo bioactivity evaluations.

## Figures and Tables

**Table 1:** List of plants selected for the study, parts used, voucher specimen numbers and extract yield values.

S.N.	Scientific Name (Family)	Local Name (Nepali)	Parts Used	Voucher Specimen No.	Extract Yield (%)
1	*A. parviflorus*	Thirjo	Whole plant	PUCD-2018-11	8.3
2	*B. asiatica*	Bhimsen-pati	Leaves	PUCD-2018-9	22.3
3	*C. papaya*	Mewa	Unripe fruits	PUCD-2018-10	4.0
4	*D. falcatum*	Nigalo	Young shoots	PUCD-2018-12	13.2
5	*S. pinnata*	Amara	Leaves	PUCD-2018-07/08	32.1

**Table 2:** Phytochemical constituents identified in different plant samples.

Phytochemical Constituents	Specific Tests	Samples				
		*A. parviflorus*	*B. asiatica*	*C. papaya*	*D. falcatum*	*S. pinnata*
Alkaloids	Mayer’s test	-	-	-	-	-
	Hager’s test	-	-	+	-	-
	Wagner test	-	-	-	-	-
Carbohydrates	Molisch’s test	-	+	+	-	-
	Benedict’s test	-	-	-	-	-
	Fehling’s test	-	+	+	-	+
Saponins	Foam test	-	+	+	-	+
Phenolic Compounds	Ferric chloride test	-	+	-	-	+
Flavonoids	Alkaline reagent test	-	+	-	-	+
Tannins	Gelatin test	-	-	+	-	-
Terpenoids	Salkowski test	+	+	+	+	+

+: Presence, -: Absence

**Table 3:** The TPC and TFC values ^a^ of the five extracts.

Sample Extract	Total Phenolic Content (mg GAE/g of Extract)	Total Flavonoid Content (mg QE/g of Extract)
*A. parviflorus*	27.48 ± 0.25	51.75 ± 2.50
*B. asiatica*	127.48 ± 1.58	648.42 ± 2.88
*C. papaya*	2.53 ± 0.25	81.75 ± 2.50
*D. falcatum*	2.43 ± 0.29	13.41 ± 1.44
*S. pinnata*	71.50 ± 1.39	425.08 ± 1.44

^a^ Values are expressed as the mean ± SD (n = 3).

**Table 4:** The IC_50_ values ^a^ for the DPPH free radical scavenging and α-amylase inhibitory activities of the extracts.

Sample	IC_50_ Values for the DPPH Free Radical Scavenging Assay (μg/mL)	IC_50_ Values for the α-Amylase Inhibitory Assay (mg/mL)
*A. parviflorus*	40.26 ± 3.44	4.76 ± 0.03
*B. asiatica*	3.04 ± 0.04	1.59 ± 0.01
*C. papaya*	41.73 ± 0.07	0.45 ± 0.02
*D. falcatum*	> 100	2.82 ± 0.05
*S. pinnata*	4.84 ± 0.12	2.11 ± 0.01
Positive control	3.16 ± 0.03	-

^a^ Values are expressed as the mean ± SD (n = 3).
